# Clinical and laboratory characteristics of COVID-19 in pregnant women

**DOI:** 10.25122/jml-2023-0044

**Published:** 2023-05

**Authors:** Oleksandra Yaroslavivna Pryshliak, Oleksandra Vasulivna Marynchak, Oksana Yevgenivna Kondryn, Ihor Hnatovych Hryzhak, Natalia Ivanivna Henyk, Oksana Mykhailivna Makarchuk, Igor Stepanovych Golovchak, Oleksandr Petrovych Boichuk, Andriy Liubomyrovych Protsyk, Mykola Valeriiovych Prokofiev

**Affiliations:** 1. Department of Infectious Diseases and Epidemiology, Ivano-Frankivsk National Medical University, Ivano-Frankivsk, Ukraine; 2. Department of Obstetrics and Gynecology named after I.D. Lanovyi Ivano-Frankivsk, National Medical University, Ivano-Frankivsk, Ukraine; 3. Precarpathian Center for Human Reproduction, Ministry of Health, Ivano-Frankivsk, Ukraine

**Keywords:** COVID-19, pregnancy, lymphocytic index, leukocyte index, disease severity prediction

## Abstract

This article discusses the distinct characteristics of COVID-19 in pregnant women and investigates potential early predictors of disease severity in this specific patient population. The study included 116 pregnant women with a confirmed diagnosis of COVID-19 in different trimesters of pregnancy. In addition to clinical features, we evaluated general clinical research methods, biochemical parameters (procalcitonin, C-reactive protein, D-dimer), and the leukocyte index of endogenous intoxication and lymphocytic index to identify potential early predictors of disease severity. All pregnant women were divided into two study groups: Group I - pregnant women with mild course, and Group II - pregnant women with moderate and severe course of COVID-19. Most pregnant women (72.4%) experienced a non-severe course characterized by catarrhal symptoms and moderate intoxication. However, pulmonary manifestations and pregnancy-related complications were detected in pregnant women from Group 2. The levels of C-reactive protein and procalcitonin in both study groups were significantly increased compared to the control group. In pregnant women with moderate and severe COVID-19, indicators of endogenous intoxication were significantly pronounced. Establishing associations between leukocyte indices and biomarkers, such as procalcitonin and C-reactive protein, enables the utilization of routine complete blood counts as a primary screening tool for predicting the severity of COVID-19 in pregnant women.

## INTRODUCTION

It is known that acute respiratory diseases pose a significant risk to pregnant women, as evidenced by the influenza pandemic in 2009. During that time, pregnant women were more likely to experience severe forms of the disease, leading to adverse effects on the course of pregnancy and an increased incidence of newborn pathologies compared to non-infected pregnant women [1,2]. Given the vulnerability of pregnant women to respiratory infections, concerns arose regarding the impact of SARS-CoV-2 infection on this population [2,3]. Pregnancy induces changes in the immune system to accommodate the development of a fetus with distinct genetic characteristics, which may impair the immune response to infections [3]. Additionally, the physiological changes during pregnancy, such as the growth of the uterus and altered coagulation factors, increase the susceptibility of pregnant women to COVID-19-related complications, particularly thromboembolic events, especially for women who are overweight and have co-morbidities such as diabetes mellitus and hypertension [3-10].

While some studies suggest that COVID-19 does not significantly impact the course of pregnancy, with outcomes similar to uninfected pregnant women [4,5], the pathogenetic mechanisms of the virus, including its effects on cells, tissues, and organs, transmission from mother to child, and perinatal consequences, are actively debated [10-13].

Furthermore, there is an ongoing accumulation of information regarding clinical and laboratory predictors of the severe course of COVID-19 in pregnant women [10,11,14]. These questions remain pertinent, even as the incidence of COVID-19 declines, highlighting the need for further investigation. This study aimed to determine the criteria for predicting severe forms of COVID-19 in pregnant women by examining the clinical and laboratory characteristics of the course of the coronavirus disease.

## MATERIAL AND METHODS

### Patient characteristics and study groups

During 2020, a total of 116 pregnant women with a confirmed diagnosis of COVID-19 were under observation and receiving treatment at the Ivano-Frankivsk Regional Perinatal Center of the Ivano-Frankivsk Regional Council. It should be noted that during that observation period, the α-variant of the SARS-COV-2 virus dominated.

The age range of pregnant women varied from 17 to 49 years, with an average age of 29.7±0.9 years. These women were at different stages of pregnancy, with 12 in the first trimester, 47 in the second trimester, and 57 in the third trimester. Most pregnant women resided in urban areas, accounting for 67.24% of the population.

Patients were divided into two study groups. Group I included 84 pregnant women with a mild course, and Group II included 32 pregnant women with an average and severe course of COVID-19. The results were compared with similar indices of the control group (CG) of pregnant women without coronavirus disease (30 people, 10 from each trimester of pregnancy). It is important to note that the high percentage of pregnant women with a mild course of COVID-19 (76.4%) was due to the early stages of the pandemic, where all pregnant women with clinical signs or a positive PCR test for SARS-CoV-2 were hospitalized as per the native algorithm. Diagnosis of the disease, determination of the severity of the course, and treatment of patients were carried out in accordance with the Order of the Ministry of Health of Ukraine dated March 28, 2020, 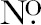 722 “Organization of medical care for patients with coronavirus disease (COVID-19)”. The inclusion criterion for the study was the presence of a confirmed case of coronavirus disease COVID-19 in a pregnant woman. The exclusion criterion was refusal to participate in the study. None of the pregnant women in the observation group were vaccinated against COVID-19 or prescribed specific anti-SARS-CoV-2 antiviral medications.

It is important to mention that this study is part of a larger research project titled “The course of infectious diseases on the background of concomitant pathology, combined chronic infections and invasions, correction of treatment.” The project has been registered under the state registration number 0119U100571 and is scheduled to be implemented from 2021 to 2023.

### Clinical evaluation and laboratory tests

Upon admission, clinical manifestations of COVID-19 were assessed, and their dynamics were monitored throughout the course of the disease. All patients underwent a PCR test to confirm the presence of the SARS-COV-2 virus on mucous membranes. Additionally, general clinical and biochemical laboratory tests were performed, and ultrasound and radiological examinations of the lungs were conducted if necessary. Special attention was given to the study of biomarkers associated with various COVID-19 complications, including procalcitonin, C-reactive protein (CRP), and D-dimer. These biomarkers have been previously identified as indicators of disease severity and inflammatory processes [6-9,14].

### Endogenous intoxication syndrome (EIS)

The widespread nature of endogenous intoxication syndrome (EIS) reactions and manifestations, resulting from the body's systemic response to inflammation, enables the use of EIS indices as criteria for assessing the severity and manifestation of the inflammatory process caused by infection. These indices also serve as prognostic markers for inflammatory complications and the progression of intoxication syndrome in various infectious diseases [15,16]. Laboratory markers such as leukocytosis, elevated erythrocyte sedimentation rate (ESR), levels of medium-mass molecules, cytokines, and specific immunoglobulins can be used to assess endogenous intoxication syndrome (EIS). However, determining some markers may not always be readily available and requires specialized equipment. As a result, it is common to characterize EIS based on hematological indices, which increases the diagnostic value of a general blood test [15,16]. Therefore, to assess the severity of COVID-19 and consider the clinical symptoms and laboratory indices recommended by the native protocol “Providing medical assistance for the treatment of the coronavirus disease (COVID-19)” dated April 02, 2020, 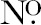 762, we used the method of calculating hematological indices of intoxication. Specifically, we considered the leukocyte index of intoxication (LII), which increases with endogenous intoxication and tissue breakdown processes, as well as the lymphocyte index (LI), which reflects the activity of two opposing pathogenetic mechanisms—systemic nonspecific inflammation and the stress-specific response of the immune system [15,16]. The dynamics of clinical symptoms and laboratory indices, the course of pregnancy, and the condition of the fetus were monitored.

### Statistical analysis

The research data was statistically analyzed using the STATISTICA-5 software on a standard personal computer. The mean values (M), the error of the arithmetic mean (m), the reliability of the differences according to the Student's t-criterion, and the correlation analysis of the interdependence between the indices, were evaluated.

## RESULTS

### Group I characteristics

Group I included 84 pregnant women with a mild course of coronavirus disease. Among them, 9 (10.7%) women were in the first trimester, 31 (36.9%) in the second trimester, and 39 (46.42%) in the third trimester. Various concomitant pathologies were found in this group, including anemia in 21 women (25.6%), gestational diabetes mellitus in 3 (3.4%), gastrointestinal tract lesions (gastritis, cholecystitis, pancreatitis) in 4 (4.5%), kidney damage (pyelitis, pyelonephritis, hydronephrosis) in 4 (4.5%), chronic arterial hypertension in 2 (2.2%), acute bronchitis in 2 (2.2%), and varicose veins of the lower limbs in 4 (4.5%) pregnant women. Among the pregnant women in Group I, prenatal screening revealed various pathological conditions. The findings included false labor occurring between 27-37 weeks of gestation in 9 (10.71%) women, threatening late spontaneous miscarriage in 7 (8.33%) women, premature labor in 1 (1.19%) woman, polyhydramnios in 3 (3.57%) women, mild preeclampsia in 4 (4.76%) women, premature placental abruption and placental pathology in 5 (5.95%) women, breech presentation in 5 (5.95%) women, intrauterine fetal development delay in 1 (1.19%) woman, and uterine scar in 3 (3.57%) women.

### Symptoms and clinical examination findings in Group I

Pregnant women in Group I presented with various symptoms upon admission, including loss of smell and taste (20.24%), general weakness (25%), nasal congestion (17.86%), sore throat (17.86%), semi-cough (5.95%) and cough (27.3%). The elevated temperature was noted in 41 (48.8%) pregnant women, but the average values did not exceed 36.9±0.05°C. Only 9.1% (8) of pregnant women had no complaints upon admission. It is worth noting that loss of smell and taste, nasal congestion, and sore throat were significantly more common in Group I compared to Group II (p<0.05). Clinical examination revealed hyperemia of the oropharynx in 57.14% of women in this group and hyper-injection of the sclera in 16.67%. Percussion over the lungs revealed unchanged lung sounds and auscultation of hard breathing was determined only in 7 (8.3%) pregnant women of this group. Shortness of breath was not reported, and the average value of SpO2 was 98±0.09%, with a respiratory rate of 19±1.02/min. Tachycardia was noted only in 2 (2.3%) women in this group and was coordinated with increased temperature.

### Imaging and laboratory findings in Group I

To assess the condition of the lungs, ultrasound examinations were performed in 69 (82.14%) women in Group I, and X-ray examinations were performed in 10 (11.9%) women. Signs of infiltrative lung changes were not found in any woman from this group, and signs of bronchitis were found in 3 (3.6%) patients.

Regarding the general blood analysis in Group I upon admission, the following average values were observed: hemoglobin level of 109.24±1.51 g/l, erythrocytes count of 3.9±0.08×10_12_/l, platelet count of 214.04±5.72×10^9^/ l, and leukocyte count of 8.65±0.40×10^9^ g/l. Further breakdown of leukocyte types revealed the following percentages: eosinophils at 1.74±0.09%, stab neutrophils at 7.23%±0.41%, segmented neutrophils at 63.17±0.56%, lymphocytes at 21.07±0.64%, and monocytes at 6.79±0.37%. ESR was 23.43±0.86 mm/hour. Comparing these indices with those of the control group, significantly higher levels of stab neutrophils and ESR (p<0.001) were found in Group I, as well as significantly lower levels of lymphocytes (p<0.05) compared to pregnant women in the control group.

### Coagulogram and biochemical indices in Group I

We analyzed the indices of the coagulogram and biochemical criteria for Group I. The average value of the prothrombin index was 99.57±1.6%, which was not significantly different from the control group (p>0.05). However, the indices of MHC and fibrinogen were significantly higher compared to the control group and were 1.13±0.01 c.u. and 3.19±0.08 g/l against 1.05±0.03 c.u. and 3. 67±0.19 g/l, respectively (p<0.001). The average indices of biochemical criteria in this group did not significantly differ from those in the comparison group (p>0.05), and their values were: ALT – 29.93±1.45 U/l, AST – 34.97±1.43 U/l, urea – 3.86±0.12 mmol/l, creatinine – 81.23±1.24 μmol/l. The C-reactive protein (CRP) level in Group I was 14.52±2.88 mg/l, and, accordingly, it was 4-fold higher than in the control group (p<0.001). The level of procalcitonin in this group was 0.21±0.03 ng/ml, which is significantly higher than in pregnant women without concomitant coronavirus disease (0.05±0.02ng/ml), (p<0.001).

### Complications and outcomes in Group I

In Group I, lower abdominal pain was detected in 10 (11.9%) pregnant women in the second trimester and 6 (7.14%) women with lower abdominal pain and bloody scanty discharge from the genital tract. Among them, 4 (4.76%) women were diagnosed with a threat of spontaneous abortion, and 1 (1.19%) woman had a miscarriage. In 6 (7.14%) women in Group I, delivery was on time, but through cesarean section. In one case, perinatal fetal death was diagnosed at 28 weeks gestation. None of the newborns in this group tested positive for COVID-19 by PCR diagnostics.

### Group II characteristics

Group II included 32 pregnant women with an average and severe course of the disease. Of these, 18.7% (6 women) were in the first trimester, 56.2% (18 women) were in the second trimester, and 31.2% (10 women) were in the third trimester. Concomitant pathologies observed in this group included anemia in 11 (34.37%) women, diabetes in 3 (9.3%) women, kidney disease in 6 (18.7%) women, cardiovascular system disease in 4 (12 .5%) women, arterial hypertension in 5 (15.6%) women, and autoimmune thyroiditis in 1 woman.

### Symptoms and clinical examination findings in Group II

In terms of clinical presentation, pregnant women in Group II reported general weakness (71.8%), nasal congestion (28.13%), sore throat (25%), main pain (9.38%), loss of taste (9.38%), and loss of smell (12.5%). An increase in body temperature to sub-febrile values (37.3°C-37.8°C) was detected in 8 (25%) women and a fever above 38.5°C – in 20 (62.5%) women. In Group II, cough was registered 3.4 times more frequently compared to Group I (90.6% vs. 27.3%, respectively) (p<0.001). Furthermore, in Group, dyspnea was observed in 14 (43.15%) women, with 8 (57.1%) requiring oxygen therapy. The average SpO2 level in this group was 97.5±0.24%, and the average respiratory rate was 23±1.52/min. Clinical examination findings in Group II revealed hyperemia of the oropharynx in 15 (46.8%) women, hyper injection of the sclera in 5 (15.6%) women, muffled lung sounds in all women, auscultatory hard breathing in 9 (28.1%) women, and weakened breathing in 23 (71.8%) women. Tachycardia was noted in 60% of women in this group and was coordinated with increased temperature.

### Imaging and laboratory findings in Group II

Ultrasound examinations were performed in all participants in Group II, and X-ray examinations were conducted in 43.75% (14 women). These diagnostic procedures revealed signs of pneumonia in all women. Upon admission, the general blood analysis of Group II showed significantly lower average values of certain indices compared to Group I. Hemoglobin levels were 1.05 times lower (104.73±1.58g/l vs. 109.24±1.51g/l, p<0.05), and platelet counts were 1.2 times lower (184.40±7×10^9^/l vs. 214.04±5.72×10^9^/l, p<0.001). However, the levels of erythrocytes (3.65±0.06×10_12_/l) and leukocytes (9.29±0.77×10^9^ g/l, of which eosinophils 1.74±0.09%, segmented neutrophils 64.20±1.07 %, lymphocytes 20.60±1.10%, monocytes 9.80±0.54%)) in Group II were not significantly different from those in Group I. The proportion of stab neutrophils was 1was significantly higher in Group II (10.27±0.80%) compared to Group I (p<0.05). In addition, the ESR was also significantly higher (27.53±0.97 mm/ h) (p<0.05).

Biochemical blood analysis revealed that ALT levels were 32.71±1.85 U/l, AST levels were 34.22±2.48 U/l, urea levels were 3.78±0.17 mmol/l, and creatinine levels were 83.43±1.97 μmol/l. However, the levels of C-reactive protein (CRP) were twice as high as in Group I (30.12±5.37 mg/l) (p<0.05). Procalcitonin levels were also three times higher (0.62±0.05 ng/ml) compared to Group I (p<0.05).

In terms of blood coagulation indices, the average values of the prothrombin index were 98.10±1.85% and did not differ from Group I. However, there were significant differences between the levels of mean platelet volume (MHC) and fibrinogen in Group II compared to Group I. The MHC levels were 1.1-fold higher in Group II (1.25±0.004 c.u.) compared to Group I (1.13±0.01 c.u.) (p<0.05), and the fibrinogen levels were 1.3-fold higher in Group II (4.22±0.16 g/l) compared to Group I (3.19±0.08 g/l) (p<0.001).

### The impact of COVID-19 on pregnant women

The course of the coronavirus disease COVID-19 with the occurrence of pneumonia in pregnant women was characterized by the activation of endogenous intoxication, which was characterized by a significant increase in LII at 84% compared to the indices of the control group (1.65±0.13 c.u. vs. 0.90±0.09 c.u., respectively) (p<0.001). This index was also 49% higher compared to pregnant women with a mild course of coronavirus disease (1.65±0.13 c.u. vs. 1.11±0.07 c.u., respectively) (p<0.001). Regarding the level of this index in pregnant women with a mild course of coronavirus disease, we found that it was not significantly increased compared to the control group (p>0.05).

### Complications and Outcomes in Group II

During the hospital stay, the following pregnancy complications were observed in Group II: 1 (3.13%) woman experienced a threat of miscarriage, 6 (18.75%) women were diagnosed with miscarriage before 37 weeks of gestation, 2 (6.25%) women with a uterine scar required medical assistance, 3 (9.36%) women delivered prematurely, and 1 woman underwent a cesarean section. It is important to note that none of the newborns in Group II tested positive for COVID-19 based on PCR diagnostics.

### D-dimer indices in pregnant women

The D-dimer index was analyzed in pregnant women with COVID-19. The findings, presented in Table 1, highlight the variations in D-dimer levels based on disease severity and gestational age.

**Table 1.** Indicators of D-dimer in pregnant women with COVID-19 depending on the severity of the disease and gestational age (M+SD)

Gestational age	Indicators of D-dimer, ng/ml			P
	Group I (n=84)	Group II (n=32)	Control group (n=30)	
I trimester	728,45+64,32 (n=9)	1276,25+105,35 (n=6)	616,06+97,4 (n=10)	pI<0,001 pI_1_>0,05 pI_2_<0,001
II trimester	1047,65+122,25 (n=31)	2943,55+455,35 (n=18)	897,45+141 (n=10)	pII<0,001 pII_1_<0,01 pII_2_<0,001
III trimester	1262,05+105,36 (n=39)	3926,36+524,2 (n=10)	1004,66+158,7 (n=10)	pIII<0,001 pIII_1_<0,001 pIII_2_<0,001

pI- p-value between group I and group II in the 1st trimester; pI_1_- p-value between the control group and group I in the 1st trimester; pI_2_- p-value between the control group and group II in the 1st trimester; pII- p-value between group I and group II in the 2nd trimester; pII_1_- p-value between the control group and group I in the 2nd trimester; pII_2_- p-value between the control group and group II in the 2nd trimester; pIII- p-value between group I and group II in the 3rd trimester; pIII_1_- p-value between the control group and group I in the 3rd trimester; pIII_2_- value between the control group and group II in the 3rd trimester;

In pregnant women with a mild course of COVID-19 (Group I), D-dimer indices during the first and second trimesters did not significantly differ from those without coronavirus disease. However, in the third trimester, D-dimer levels were significantly higher in Group I compared to the control group (pIII^1^<0.001). In pregnant women with a more severe course of COVID-19 (Group II), D-dimer indices increased with advancing gestational age and were significantly higher than in pregnant women without coronavirus disease and those with a mild course of the disease at all stages of pregnancy. However, no signs of thrombotic complications were found in pregnant women with coronavirus infection with high D-dimer indices. It should also be noted that to prevent venous thromboembolism, pregnant women with a moderate and high risk of occurrence were routinely prescribed low-molecular-weight heparins, regardless of the coronavirus disease. There were 32 (38%) pregnant women in the study group with a mild course, 32 (100%) women with a medium-severe and severe course of COVID-19, and 13 (43.3%) pregnant women in the control group. This may have influenced the absence of thrombotic complications.

### Leukocyte Intoxication Index (LI)

A significant decrease in LI was observed in both studied groups compared to the control one. The quantitative values of this index were 0.03±0.01 in Group I and 0.23±0.03 in Group II (p<0.001). However, it is worth noting that in pregnant women who developed pneumonia due to COVID-19, LI was 1.3-fold lower compared to Group I (p<0.05).

In Group I, which had a mild course of COVID-19, no correlational interdependence was found between the leukocyte indices of the general blood test and the level of biomarkers of the severe course of the disease. In Group II, a direct correlation of medium strength was found between the level of leukocyte intoxication index (LII) and procalcitonin (r=+0.39, p<0.05) and a weak correlation between LII and CRP (r=+0.27, p<0.05. Furthermore, the inverse correlation between LI and the level of procalcitonin (r=-0.62, p<0.05) and CRP (r=-0.39, p<0.05) is of medium strength.

## DISCUSSION

The initial phase of diagnosis and treatment of coronavirus infection, when all cases of the disease were subjected to hospitalization, showed that the non-severe course of COVID-19 prevails among pregnant women (72.4% with a mild course), which is confirmed by the data of most researchers [17-20]. Additionally, it was noted that most hospitalized pregnant women with COVID-19 were in the second and third trimesters (89.66%). This trend was consistent across both groups of pregnant women, irrespective of the disease severity, suggesting increased susceptibility to COVID-19 starting from the second trimester.

In 10 pregnant women (31.25%) with moderately severe and severe course of the coronavirus disease, clinical symptoms and the results of general clinical laboratory tests upon admission to the hospital did not differ from the average indices of pregnant women in the group of patients with a mild course of COVID-19. It was only after 5-7 days that signs of a severe course of the disease appeared. In the group with a severe COVID-19 coronavirus disease, the percentage of pregnant women with concomitant somatic and prenatal pathology was higher than in the group with a mild course of the disease [20-24].

The mild course of COVID-19 in pregnant women was clinically characterized by a pronounced catarrhal syndrome, slightly pronounced intoxication phenomena, the absence of pneumonia, and other complications of the coronavirus disease. The general blood analysis revealed significantly higher levels of stab neutrophils, elevated ESR, and lower lymphocyte levels compared to pregnant women without coronavirus infection. Significantly higher levels of CRP and procalcitonin were found than in pregnant women without concomitant coronavirus disease. Moreover, D-dimer levels in pregnant women of Group I varied depending on the gestational age, with higher values observed in the third trimester compared to the control group.

In pregnant women with moderately severe and severe COVID-19, the clinical picture was characterized by pneumonia (cough, shortness of breath, oxygen dependence) in the early period of the disease. In the general blood test, significantly lower levels of hemoglobin and higher levels of stab neutrophils and ESR were determined, compared to similar indices in pregnant women with COVID-19 with a mild course of the disease.

Pregnant women in Group II, who had a severe course of COVID-19, showed significantly higher levels of CRP and procalcitonin compared to pregnant women in Group I, who had a mild course of the disease. These biomarkers, associated with disease severity, were elevated in Group II compared to Group I. In Group II, D-dimer indices increased with gestational age and were significantly higher than similar indices in pregnant women with mild coronavirus disease (1.7-fold during the 1st trimester, 2.8-fold in the 2nd trimester, and 3.1-fold in the 3rd trimester of pregnancy, p<0.001). However, no signs of thrombotic complications were found in pregnant women with coronavirus infection with high D-dimer levels. It is worth noting that low-molecular-weight heparins were routinely prescribed to pregnant women at moderate to high risk of venous thromboembolism, regardless of their COVID-19 status [5,8,9,13,22,25]. There were 32 such pregnant women in the study group (38%) with a mild course, 32 (100%) with a moderately severe and severe course of COVID-19, and 13 (43.3%) among pregnant women in the control group. Perhaps this is what influenced the absence of thrombotic complications.

Pathological processes induced by coronavirus infection during pregnancy can lead to perinatal complications such as miscarriages, premature births, and false contractions [18,20-22]. Our study confirmed the potential risk of such perinatal consequences in pregnant women with COVID-19.

During the second trimester, 11.4% of pregnant women in this group experienced lower abdominal pain. Additionally, 11.9% of pregnant women in the second trimester reported lower abdominal pain, and 7.14% experienced lower abdominal pain and bloody scanty discharge from the genital tract. Among them, 4.76% of pregnant women were diagnosed with a threat of spontaneous abortion, and 1.19% of pregnant women had a miscarriage.

During hospitalization, 3.13% of women were at risk of miscarriage, 18.75% of women were diagnosed with miscarriage before 37 weeks of gestation, 6.25% received medical care with a uterine scar, 9.36% of women had premature births, and cesarean delivery was performed in 3.13% of women in Group II. The results of our study indicate a higher prevalence of gynecological pathology during pregnancy among pregnant women with moderately severe and severe COVID-19, aligning with findings reported by other researchers [10,11,18]. However, it is important to note that the observed difference was not statistically significant (p>0.05), and the prenatal history of pregnant women in this group was also more severe. Therefore, this aspect needs additional research.

In our study, none of the newborns showed clinical signs of COVID-19, hypoxic manifestations, or tested positive for the SARS-CoV-2 virus through PCR diagnostics. These results align with findings from other researchers, although it does not entirely rule out the possibility of transplacental transmission [12,26-28]. Studies have highlighted the differential impact of COVID-19 on pregnancy outcomes at different gestational ages [20,29,30]. When analyzing the medical history, we noted that most respiratory symptoms were observed in women in the second trimester of pregnancy. This phenomenon may be attributed to rapid uterine growth, increased blood volume, and heightened cardiovascular burden during this period, potentially making the disease more severe. However, some studies have reported a high incidence of pregnancy complications in the third trimester [31].

Additionally, a high rate of cesarean section deliveries (approximately 50%) has been reported among pregnant women with COVID-19 worldwide [28-30]. According to our research, delivery was performed on time via cesarean section in 7.14% of women in Group I and 3.13% of pregnant women in Group II. These findings emphasize the need for further research to elucidate the potential pathological mechanisms underlying the impact of the virus on the placental system. Perinatal complications may be associated with possible physiological immunosuppression in pregnant women and extragenital pathology, requiring further investigation.

The progression of COVID-19 and the occurrence of various complications and consequences are attributed to a complex interplay of immunopathological and inflammatory reactions, cytokine cascades, endothelial dysfunction, and coagulation disorders, all of which are associated with the underlying coronavirus infection [10, 23-26]. Consequently, there is considerable interest among researchers to identify early predictors of disease severity, particularly in pregnant women. Today, various studies have highlighted the role of neutrophils, leukocytes, platelets, and monocytes in COVID-19 pathogenesis and its associated complications [4, 32-34]. On the other hand, determining the level and ratio makes it possible to use these indices to predict the expressiveness of pathological processes.

The analysis of scientific publications confirms the interest in determining the value of diagnostic indices for predicting morbidity in pregnant women. For instance, a study involving 498 pregnant women established the high sensitivity of determining the absolute number of lymphocytes and the ratio of neutrophils to leukocytes in predicting the progression of severe COVID-19 in pregnant women and potential perinatal complications [32]. In another study, which included 2,649 pregnant women, significantly higher levels of the immune-inflammatory index (neutrophil platelet)/lymphocyte) and the immune response index (neutrophil monocyte)/lymphocyte) were determined in pregnant women with coronavirus in women and perinatal pathology [33]. Moreover, there are also reports on the potential investigation of neutrophil/lymphocyte (NLR) and platelet/lymphocyte (PLR) ratios in pregnant women with COVID-19 [34]. The authors found higher levels of PLR in pregnant women with a severe course of COVID-19 and existing perinatal pathology and high levels of NLR in all pregnant women with COVID-19, regardless of the disease severity.

We determined that in pregnant women with signs of pneumonia, the LII index was significantly higher, and the LI index was significantly lower than the similar indices, both in the group of pregnant women without coronavirus disease and in the group of pregnant women with a mild course of COVID-19. The results obtained indicate that the course of the COVID-19 disease in pregnant women was accompanied by the activation of endogenous intoxication, the processes of tissue decay, and the occurrence of an immunodeficiency condition by the cell type, in particular, a decrease in non-specific anti-infective protection, which was more pronounced when a complication in the form of pneumonia occurred [2, 3, 12].

Given the high sensitivity of these indices and their ease of use, these indicators should be used as early predictors of the severity and prediction of prenatal complications in pregnant women with COVID-19. Further exploration of the influence of the disease on pregnancy and its outcomes warrants investigation into the specific characteristics of pregnant women who have received the COVID-19 vaccine compared to those who have not. It is especially important to examine the dynamics of immune response indices, with a particular focus on LI.

Continuing the study of the features of the influence of the disease on the course and consequences of pregnancy, it seems promising to study their features in pregnant women vaccinated against COVID 19 compared to those not vaccinated, especially the dynamics of immune response indices, in particular LI [35].

## CONCLUSION

Our study revealed that the majority (73%) of pregnant women affected by COVID-19 did not experience a severe course of the disease. However, pregnant women entering the second trimester are considered a high-risk group for SARS-CoV-2 infection. Moreover, those with preexisting somatic and prenatal conditions are particularly vulnerable to a more severe disease course. The clinical presentation of mild COVID-19 was typically characterized by catarrhal syndrome and moderate intoxication. In contrast, the moderately severe and severe courses exhibit early onset of pronounced symptoms of intoxication and lung damage. However, approximately 31% of pregnant women with a moderate or severe course developed symptoms of lung damage 5-7 days after the disease onset.

Furthermore, our study confirms the significance of procalcitonin and C reactive protein as biomarkers for predicting the severe course of coronavirus infection. However, the elevated D-dimer indices observed in pregnant women with moderately severe and severe disease cannot be used to predict possible fibrinous disorders in this category of patients. The correlations between leukocyte indices and biomarkers of disease severity suggest that a general blood test can serve as an initial screening tool to predict the severe course of COVID-19 in pregnant women.
